# Targeting the Extracellular Signal-Regulated Kinase 5-Cellular Jun-Vimentin Axis to Inhibit Epithelial-Mesenchymal Transition and Metastasis in Patients with Triple-Negative Breast Cancer

**DOI:** 10.7150/ijms.131682

**Published:** 2026-06-17

**Authors:** Chia-Chi Chen, Shu-Jyuan Chang, Cheng-Loong Liang, Hieu D. H. Nguyen, Chi-Wen Luo, Yi-Zi Chen, Yu-Tzu Yang, Mei-Chiang Hsu, Sin-Hua Moi, Chao-Ming Hung, Mei-Ren Pan

**Affiliations:** 1 Graduate Institute of Clinical Medicine, Kaohsiung Medical University, Kaohsiung 807, Taiwan.; 2 Department of Pathology, E-Da Hospital, I-Shou University, Kaohsiung 824, Taiwan.; 3 School of Medicine, College of Medicine, I-Shou University, Kaohsiung 824, Taiwan.; 4 Department of Neurosurgery, E-Da Hospital, I-Shou University, Kaohsiung 824, Taiwan.; 5 Division of Breast Oncology and Surgery, Department of Surgery, Kaohsiung Medical University Hospital, Kaohsiung 807, Taiwan.; 6 Department of Surgery, E-Da Cancer Hospital, I-Shou University, Kaohsiung 824, Taiwan.; 7 Drug Development and Value Creation Research Center, Kaohsiung Medical, University, Kaohsiung 807, Taiwan.

**Keywords:** ERK5, TNBC, epithelial-mesenchymal transition, metastasis, Jun, Vimentin

## Abstract

Breast cancer is the second most common cancer worldwide and remains the leading cause of cancer-related deaths among women. Triple-negative breast cancer (TNBC) represents approximately 15-20% of all breast cancer cases and is characterized by an aggressive clinical course and a high risk of metastasis. Extracellular signal-regulated kinase 5 (ERK5) is a critical biomarker that promotes tumor progression through mechanisms involving cell proliferation, invasion, and metastasis; however, its precise role in epithelial-mesenchymal transition (EMT) in TNBC remains unclear. In this study, we analyzed data from 117 patients with TNBC and found that high ERK5 expression was significantly associated with tumor progression, shorter progression-free survival, and shorter overall survival. RNA sequencing of a highly metastatic TNBC cell line revealed that ERK5 knockdown modulated the expression of various gene clusters, particularly those associated with DNA repair, G2/M checkpoint regulation, and angiogenesis. In addition, ERK5 knockdown in a mouse xenograft model significantly suppressed tumor proliferation and lung metastasis, inhibited tumor cell migration, and reduced the expression of EMT-related proteins. Mechanistically, our data further demonstrated that ERK5 regulates the interaction between cellular JUN (c-JUN) and the vimentin promoter, thereby modulating vimentin expression and downstream signaling pathways. A significant positive correlation between ERK5 and vimentin expression in human TNBC tissue specimens further supported this regulatory association. These findings suggest that ERK5 mediates the recruitment of c-JUN to regulate vimentin expression, thereby promoting EMT and metastasis. Thus, the ERK5/c-JUN/vimentin axis may be a potential therapeutic target to improve clinical outcomes in patients with TNBC.

## Introduction

According to the latest Global Cancer Observatory statistics from the International Agency for Research on Cancer, approximately 20 million people were newly diagnosed with cancer in 2022, and an estimated 9.7 million deaths were attributed to cancer during the same year. Breast cancer is the second most commonly diagnosed cancer worldwide, following lung cancer, but remains the most prevalent cancer among women. Notably, breast cancer is the leading cause of cancer-related deaths among women in more than half of all countries globally 1. In the United States, the incidence of breast cancer in women has gradually increased at an annual rate of approximately 0.6% since the mid-2000s. This increase is more pronounced among women < 50 years, with an annual increase of 1.1%, compared with 0.5% in women aged ≥ 50 years 2. Another study suggests that by 2040, the global incidence of breast cancer may increase by approximately 31% compared with 2020 estimates 3. These data indicate that breast cancer remains a critical global public health challenge.

In clinical practice, breast cancer subtypes are classified based on the expression of hormone receptors, including estrogen receptor (ER) and progesterone receptor (PR), as well as human epidermal growth factor receptor 2 (HER2) and the Ki-67 proliferation index, which guide therapeutic decision-making. Breast cancer cells lacking the expression of these three biomarkers are categorized as triple-negative breast cancer (TNBC), accounting for approximately 15-20% of all breast cancer cases 4. TNBC is associated with a higher risk of distant metastasis and poorer overall prognosis than other subtypes. Due to the absence of specific biomarkers, treatment for TNBC largely relies on surgical resection, adjuvant chemotherapy (e.g., docetaxel, doxorubicin, cyclophosphamide, paclitaxel, and carboplatin), and radiation therapy. Despite advancements in medical technology and research, current National Comprehensive Cancer Network guidelines indicate that conventional chemotherapy remains the primary systemic treatment for TNBC 5. Targeted therapies, such as Poly (ADP-ribose) polymerase inhibitors (e.g., olaparib) and immune checkpoint inhibitors (e.g., pembrolizumab), are currently limited to select patient subpopulations that meet specific clinical criteria. Nevertheless, many patients with TNBC continue to experience treatment resistance, distant metastasis, and disease recurrence, highlighting the need for more effective therapeutic options.

Extracellular signal-regulated kinase 5 (ERK5), also known as mitogen-activated protein kinase 7 (MAPK7) or big mitogen-activated protein kinase 1 (BMK1), is the most recently identified member of the MAPK family, which also includes ERK1/2, p38 MAPK, and c-Jun N-terminal Kinase (JNK)1/2/3. ERK5 is an 816-amino-acid protein encoded by the MAPK7 gene and has a molecular weight approximately twice that of other MAPK family members, earning the designation BMK1. The N-terminal kinase domain of ERK5 shares 66% sequence homology with ERK2, whereas the C-terminal region domain includes a unique transcriptional activation domain with autophosphorylation capability and a nuclear localization signal 6-8. ERK5 is widely expressed in mammalian tissues and regulates numerous physiological and pathological processes by modulating diverse downstream effectors and signaling pathways. Previous studies identified several transcription factors downstream of ERK5, including activator protein 1 (AP-1), cellular FBJ murine osteosarcoma viral oncogene homolog (c-FOS), cellular myelocytomatosis viral oncogene homolog (c-MYC), twist family BHLH transcription factor (Twist), nuclear factor kappa beta (NF-κB), and myocyte enhancer factor 2 (MEF2) 9-13. Although early research primarily implicated ERK5 in embryonic angiogenesis and endothelial cell survival 6, 7, more recent evidence indicates that ERK5 also contributes to tumor cell growth, local tissue invasion, distant metastasis, metabolic reprogramming, cancer stem cell properties, and chemoresistance development 10, 14-19, consistent with updated cancer hallmarks described in 2022 20.

EMT is a complex biological process in which epithelial cells lose their defining features, such as intercellular junctions, cell polarity, and quiescence, while acquiring mesenchymal traits, including cytoskeletal reorganization, enhanced motility, and invasive capacity 21. EMT occurs during physiological processes, including embryogenesis, organogenesis, and wound healing, and is also a central driver of tumor invasion and distant metastasis. EMT is closely correlated with cancer stem cell-like properties, which enhance tumor cell self-renewal capacity and cellular plasticity, thereby accelerating tumor progression 14, 22, 23. Previous studies have demonstrated that ERK5 promotes EMT in breast cancer by increasing the expression of transcription factors such as Snail2 and zinc-finger E-box-binding homeobox 1 (ZEB1), thereby facilitating distant metastasis. In murine TNBC models, ERK5 inhibition resulted in increased E-cadherin expression, reduced circulating tumor cells, and decreased lung metastasis 10, 24, 25.

In 2015, Zhai *et al*. discovered that miR-143 directly targets the 3'-untranslated region of ERK5 in hormone-positive breast cancer, thereby suppressing ERK5 expression and its downstream regulation of the glycogen synthase kinase 3 beta (GSK-3β)/Snail signaling pathway 22. This modulation increased epithelial-associated biomarkers, such as E-cadherin and zonula occludens-1, and reduced mesenchymal-associated genes, including vimentin and N-cadherin. It also inhibited tumor cell migration, invasion, and growth rate. Additionally, Pavan *et al*. demonstrated that inhibition of the MEK5-ERK5 signaling pathway in murine TNBC 4T1 cells completely reversed transcription growth factor beta (TGF-β)-induced EMT and suppressed tumor cell migration and invasion in both 4T1 and human MDA-MB-231 TNBC cells 23. Similarly, Bhatt *et al*. revealed that MEK5/ERK5 inhibition impaired EMT maintenance and migratory capabilities in MDA-MB-231 cells 24.

These studies suggest that ERK5 may play a crucial role in regulating EMT and influence TNBC progression and patient prognosis. However, its precise mechanistic function in TNBC remains insufficiently defined. In this study, we integrated analyses of online cancer databases, clinical TNBC specimens, human TNBC cell lines, and murine TNBC models to investigate the impact of ERK5 on EMT-related protein expression and elucidate the potential underlying molecular mechanisms.

## Material and methods

### Cell culture and RNA interference

The human triple-negative breast cancer cell line IV-2-1, characterized by a high propensity for lung metastasis, was used in this study and kindly provided by Professor Lu-Hai Wang (China Medical University, Taiwan). Additionally, the 4T1 TNBC cell line was obtained from the American Type Culture Collection (CRL-2539, ATCC). All cells were cultured in Dulbecco's modified Eagle's medium supplemented with 10% fetal bovine serum, 100 U/mL penicillin, and 100 µg/mL streptomycin (Gibco).

ERK5 knockdown was achieved using plasmid-based short hairpin RNA (shRNA) targeting ERK5. These shRNAs produce mature small interfering RNA molecules that can bind to ERK5 mRNA, thereby blocking its subsequent translation into protein and expression. All shRNAs were purchased from the National RNAi Core Facility (Academia Sinica, Taiwan). A stable knockdown cell population was selected with puromycin (P8833, Sigma-Aldrich). The efficacy of ERK5 knockdown was further confirmed using western blot analysis, which verified the successful depletion of ERK5 in these cell lines.

### Drug treatment and wound healing assay

To assess cellular migratory capacity, wound-healing (scratch) assays were performed using 4T1 control and ERK5-knockdown 4T1 TNBC cell lines. Cells were seeded into an Ibidi Culture-Insert 2 Well system at 3×10^5^ cells/mL and incubated at 37 °C with 5% CO₂ for 24 hours. For drug treatment, cells were exposed to the ERK5 inhibitor BIX 02189 (10 μM; S1531, Selleck Biotechnology Limited) before incubation. After cell attachment, the insert was then carefully removed from the wells using sterile forceps. Fresh medium was added, and cultures were incubated for an additional 17-24 h at 37 °C with 5% CO₂. The wound width was imaged at 0 and 17 h to assess cell migration.

### Western blotting

After total protein extraction from the cells, protein concentrations were determined, and equal amounts of protein were loaded into each lane. β-actin was used as a loading control. Proteins were separated by SDS-PAGE using stacking and separating gels of appropriate concentrations, then transferred to polyvinylidene difluoride (PVDF) membranes. After blocking, the membranes were incubated with the indicated primary antibodies, followed by secondary antibodies (Jackson ImmunoResearch, Cambridgeshire, UK). Protein signals were visualized using enhanced chemiluminescence (ECL; PerkinElmer, Waltham, MA, USA) and recorded using a ChemiDoc system (Bio-Rad). The following primary antibodies were used: human ERK5 antibody (#3372, Cell Signaling Technology), mouse ERK5 antibody (#3372, Cell Signaling Technology), mouse E-cadherin antibody (#3195, Cell Signaling Technology), mouse Slug antibody (GTX12192), mouse N-cadherin antibody (33-3900, Thermo Fisher Scientific), mouse vimentin antibody (ab8978, Abcam), mouse ZEB1 antibody (NBP1-05987, Novus Biologicals), and mouse β-catenin antibody (#3700, Cell Signaling Technology).

### RNA sequencing and data analysis

RNAi was used to knockdown ERK5 expression in the human TNBC cell line MDA-MB-231-IV-2-1. Total RNA was extracted from both experimental and control groups using the TRIzol Reagent (15596026, Thermo Fisher Scientific). The extracted RNA was then sent to AllBio Science Incorporated for high-throughput sequencing and preliminary analysis to identify potential functional gene groups and upstream and downstream pathways influenced by ERK5 expression. Based on the sequencing results, further analyses were conducted using Ingenuity Pathway Analysis and Gene Set Enrichment Analysis (GSEA). For GSEA analysis, we used the criteria of NES≥ 1.0, NOM p-value ≤ 0.05, and FDR q-value ≤ 0.25 to identify gene sets that showed significant changes in expression following ERK5 knockdown. These analyses were complemented by literature reviews and online database searches to identify candidate proteins and signaling pathways for subsequent testing and validation. Finally, Western blotting was performed to validate the sequencing data.

### Chromatin Immunoprecipitation (ChIP)

ChIP assays were then performed to evaluate c-JUN binding to the vimentin promoter (Figure 5E and Figure 5F). The TNBC cells from both the experimental and control groups were lysed, and chromatin was sonicated to produce 200-1000 bp fragments. Next, samples were incubated with c-JUN-targeting antibody (1:50; #9165, Cell Signaling Technology). DNA fragments bound to the c-JUN transcription factor were isolated from the total pool of DNA fragments. The samples were then treated with proteinase K to dissociate c-JUN from the DNA fragments, thereby removing the protein and leaving only the DNA. Finally, promoter fragments near the -700 bp region of the vimentin gene were amplified and quantified by quantitative real-time polymerase chain reaction (qPCR).

### Quantitative real-time polymerase chain reaction (qPCR)

Total RNA was extracted using the TRIzol Reagent (15596018, Thermo Fisher Scientific), followed by complementary DNA (cDNA) synthesis using specific primers and SuperScript III reverse transcriptase (Invitrogen). The synthesized cDNA was used as a template for subsequent PCR amplification and quantitative analysis using a Roche LightCycler 480 II system (Basel). The primers used in this study were as follows: mouse vimentin (NM_011701) (forward, 5'-AGGAGGCCGAGGAATGGT-3' and reverse, 5'-CATCGTTGTTCCGGTTGG-3'), mouse GAPDH (NM_008084) (forward, 5'-TTCCATCCTCCAGAAACCAG-3' and reverse, 5'-CCCTCGAACTAAGGGGAAAG-3'), and mouse AP-1-Chip (forward, 5'-TCGGACAATGAATGAGCTTAGC-3' and reverse, 5'-ACTGCCTCTGGAGTAAAGCC-3').

### Online databases

Breast cancer-related datasets were utilized from online databases, including TIMER2.0 (https://timer.cistrome.org/) and KM-plotter (https://kmplot.com/). The following filters were applied: ER status (immunohistochemistry: ER-negative), array (ER-negative + PR status, IHC: PR-negative), and HER2 status array (HER2-negative). These filters were used to identify TNBC cases for final inclusion. The Ingenuity Pathway Analysis database (https://digitalinsights.qiagen.com/) was utilized to analyze the RNA sequencing data. Additionally, data from the Eukaryotic Promoter Database (EPD, https://epd.expasy.org/epd/) were used to predict potential c-JUN (FOS: JUN) binding sites in the upstream region (-1000 to +100 bp) of the vimentin promoter.

### Clinical specimens from TNBC patients

In this study, a total of 117 specimens were collected from patients diagnosed with TNBC between 2009 and 2019 from E-Da Healthcare Group Hospitals and Kaohsiung Medical University Chung-Ho Memorial Hospital. All samples were anonymized and analyzed using immunohistochemical (IHC) and hematoxylin and eosin (H&E) staining for histopathological evaluation. Comparative analyses were performed based on various clinical factors, including pathological stage, tumor size, use of radiotherapy and chemotherapy, recurrence, and overall survival. All experimental procedures were conducted under the supervision and approval of the Institutional Review Board of E-Da Healthcare Group hospitals and Kaohsiung Medical University Chung-Ho Memorial Hospital (EMRP-111-141 and KMUHIRB-F(I)-20200107).

### Immunohistochemistry

We evaluated protein expression in tumor cells from clinical patient samples and in murine tissues using the H-score system 25, 26. Immunohistochemical staining was independently evaluated by two investigators blinded to the patients' clinical information, using the predefined scoring criteria described below. In brief, after deparaffinizing the 4 μm tissue sections with xylene, the sections were rehydrated through a graded series of alcohols, followed by washing with Tris-buffered saline with Tween 20. The tissue sections were incubated with primary antibodies targeting human or mouse proteins. The following antibodies were used: human ERK5 (1:100; sc-398015, Santa Cruz Biotechnology), human vimentin (1:2000; GTX100619, GeneTex), mouse ERK5 (1:100; sc-398015, Santa Cruz Biotechnology), mouse vimentin (1:1000; GTX100619, GeneTex), mouse E-cadherin (1:100; 610182, BD Biosciences), mouse Slug (1:500; GTX121924, GeneTex), mouse C-JUN (1:50; #9165, Cell Signaling Technology, Inc.), and mouse Ki-67 (1:50; #12202, Cell Signaling Technology, Inc.). Immunohistochemical staining was subsequently performed using the Dako EnVision Detection System (K5007, Peroxidase/DAB, Rabbit/Mouse, Agilent Technologies, Inc.) to detect the target proteins. Subsequently, tissue sections were dehydrated with alcohol, mounted on slides, and observed under a microscope for thorough analysis and interpretation.

The scoring process involved selecting 10 random high-power fields (400× magnification) within the tumor cell region. For each field, the percentages of tumor cells staining 0 (no expression), 1 (weakly positive), 2 (moderately positive), and 3 (strongly positive) were calculated. Both nuclear and cytoplasmic staining results were considered positive. The final H-score was determined by averaging the results from the 10 high-power fields, calculated using the following formula: H-score = (1 × [% of cells with intensity 1]) + (2 × [% of cells with intensity 2]) + (3 × [% of cells with intensity 3]), yielding a score between 0 and 300. The optimal cut-off values for ERK5 protein expression were determined using the X-tile algorithm based on disease-free survival (DFS) 27. Based on these optimal cut-off points, ERK5 H-scores were classified into high-, medium-, and low-expression groups.

To assess E-cadherin expression in tumor cells from mouse breast cancer tissues, we evaluated the overall membrane staining intensity and assigned scores of 0, 1, 2, and 3 for no, weak, moderate, and strong expression, respectively. Additionally, the percentage of tumor cells exhibiting membrane staining, including both complete and partial, was quantified and included in our analysis. For Slug, vimentin, Ki-67, and c-JUN expression in tumor cells from both clinical patient samples and mouse tissues, the average staining intensity within either the nucleus or cytoplasm was examined using the same scoring system (0, 1, 2, and 3 for no, weak, moderate, and strong expression, respectively) and the percentage of positive tumor cells. The IHC score was calculated as the product of the percentage of tumor cells showing positive staining and the staining intensity. These metrics informed our subsequent analysis.

### Orthotopic BALB/c mouse model

Experiments involving animals were approved by, and conducted in accordance with, the guidelines and regulations of the Institutional Animal Care and Use Committee of Kaohsiung Medical University, Kaohsiung, Taiwan (IACUC no. 111088). In this study, eight immunocompetent BALB/c mice were obtained from the National Laboratory Animal Center and randomly assigned to either the control or experimental group, with four mice per group. The 4T1 mouse TNBC cell line was selected for the *in vivo* experiments. Following established protocols from previous studies, 1×10^4^ 4T1 cells with ERK5 knockdown (via RNAi) and control 4T1 cells were orthotopically injected into the fourth mammary fat pad of each group using a 27-gauge needle. Tumor progression and metastatic spread were monitored weekly using an IVIS imaging system at the Laboratory Animal Center of Kaohsiung Medical University. After 4 weeks, the mice were euthanized using carbon dioxide inhalation, and the sizes and numbers of primary mammary tumors and lung metastases were recorded. Tumors from both the primary and metastatic sites were harvested, fixed in neutral formalin, and processed into paraffin-embedded blocks and sections. These sections were then subjected to standard H&E staining and immunohistochemical analysis.

### Statistical analysis

The SPSS statistical software (version 19.0; IBM Corp.) was used to analyze the experimental results and related data. Student's *t*-test was used to assess differences in expression between the two sample groups. To analyze protein expression, clinical parameters, and pathological characteristics in samples from patients with TNBC, we used chi-square tests, Pearson's correlation, and Kaplan-Meier survival analysis. Statistical significance in all analyses was defined as a *p*-value < 0.05.

## Results

### Elevated ERK5 expression in triple-negative breast cancer is associated with poor prognosis

**Figure 1:**
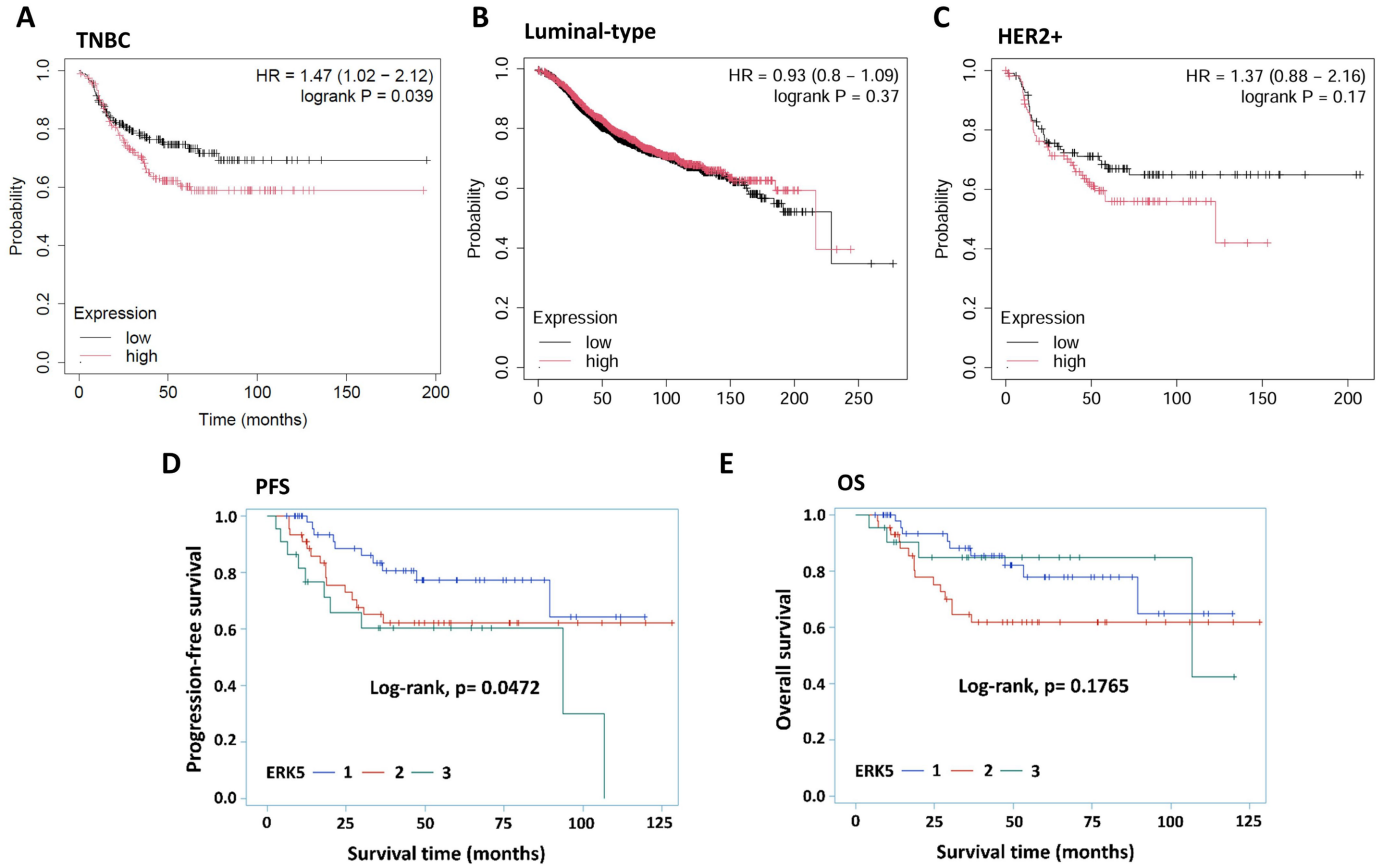
**ERK5 is highly expressed in triple-negative breast cancer** (**TNBC) and is associated with poor prognosis.** (A-C) Analysis using messenger RNA (mRNA) expression data (207292.s.at) from the Kaplan-Meier plotter online database. Correlation of ERK5 expression in human tumor tissue with (D) progression-free survival (PFS) and (E) overall survival (OS).

First, we analyzed ERK5 expression using the Kaplan-Meier plotter (KM-plotter) online database 30, which leverages gene chip data to assess messenger RNA (mRNA) expression (207292.s.at). A total of 392 patients with TNBC were included in the analysis. Our results indicated that high ERK5 expression was significantly associated with shorter relapse-free survival (RFS) in patients with TNBC (*p* < 0.05). In contrast, this correlation was not observed in patients with luminal-type or HER-2 positive breast cancer (Figure 1A-1C). By contrast, ERK5 expression was not significantly associated with overall survival (OS) (n=153, *p* = 0.14) or distant metastasis-free survival (DMFS) in TNBC patients (n=306, *p* = 0.22) (Supplementary Figures 1A and 1B).

We next validated these findings using clinical TNBC tumor specimens. In total, 117 TNBC samples diagnosed between 2009 and 2019 were collected from E-Da Healthcare Group Hospitals and Kaohsiung Medical University Chung-Ho Memorial Hospital. The clinicopathological parameters and their associations with ERK5 expression are presented in Table 1. The samples were subjected to routine hematoxylin and eosin and ERK5 IHC staining for interpretation and analysis. ERK5 expression levels in tumor cells were evaluated using the H-score by two independent investigators. Our findings demonstrated a significant negative correlation between ERK5 expression (H-score) in TNBC tumor cells and progression-free survival (PFS) (Figure 1D, p < 0.05).

In contrast, no statistically significant association was observed between ERK5 expression and OS (Figure 1E). Similar to Figure 1D, Pearson's correlation analysis revealed that ERK5 expression was significantly associated with recurrence and tumor progression (p < 0.05) (Table 2). Moreover, Univariate Cox regression analysis showed that high ERK5 expression, tumor size, T stage, M stage, and dermal invasion were significantly associated with PFS. After adjustment for clinicopathological covariates in the multivariable model, high ERK5 expression remained significantly associated with poorer PFS (HR = 2.875, 95% CI = 1.152-7.174, p = 0.0236), suggesting that high ERK5 expression is an independent prognostic factor for PFS in TNBC patients (Table 3).

### ERK5 knockdown reprograms immune, DNA repair, cell cycle gene, and angiogenesis sets

**Figure 2:**
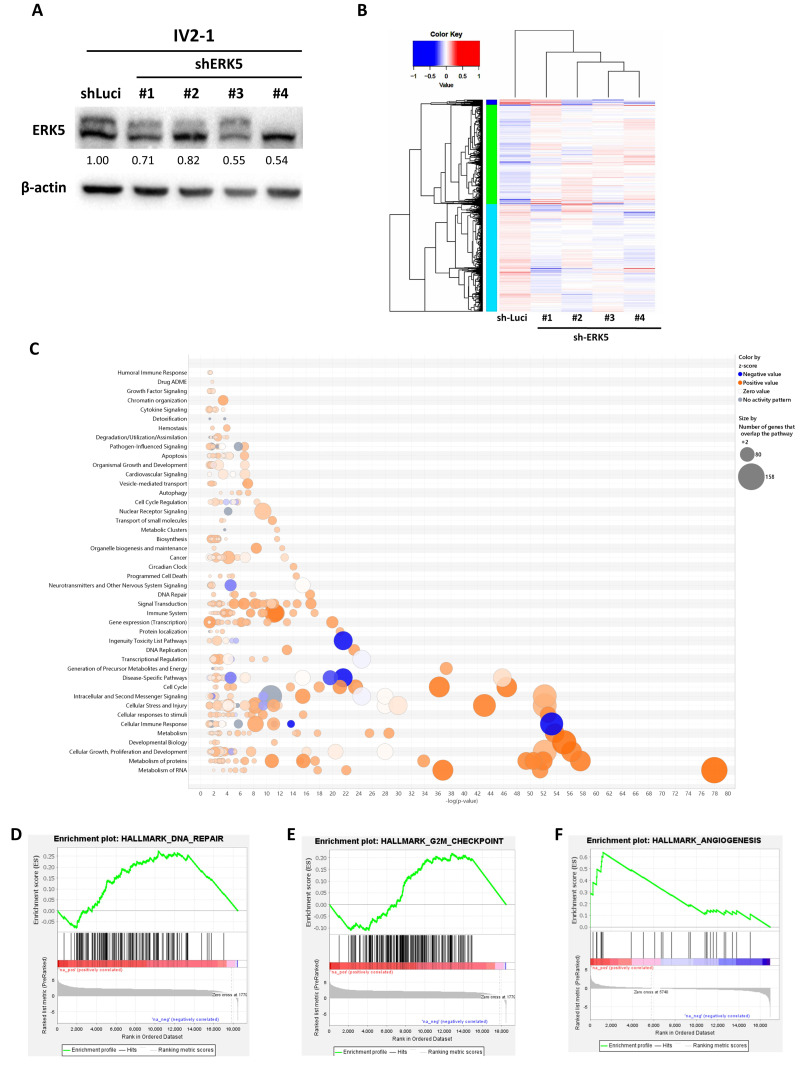
** ERK5 in TNBC regulates genes associated with immunity, DNA repair, cell cycle progression, and angiogenesis.** (A) Western blot analysis confirming the successful establishment of stable ERK5-knockdown TNBC cell lines. β-actin was used as a loading control. (B) RNA sequencing results visualized as a heatmap. (C-F) RNA sequencing data were further analyzed using Ingenuity Pathway Analysis and Gene Set Enrichment Analysis (GSEA) to identify differentially expressed key pathways in human TNBC tumor cells.

To investigate the functional consequences of ERK5 inhibition, four distinct shRNAs were used to generate stable ERK5 knockdown in a human TNBC cell line with a high propensity for lung metastasis (IV2-1-shERK5 #1-4) (Figure 2A). We then performed RNA sequencing (RNA-seq) on ERK5-knockdown human TNBC cell lines (IV2-1 shERK5 #1-4) and control tumor cell lines (IV2-1 shLuci), and generated a heatmap to visualize transcriptional changes (Figure 2B). Differentially expressed genes were further analyzed using Ingenuity Pathway Analysis and GSEA of RNA-seq data from the experimental and control groups. The results revealed that inhibition of ERK5 expression in human TNBC cell lines significantly altered the expression of immune-response genes compared with the control group (Figure 2C). In addition to immune-related pathways, ERK5 knockdown also led to significant changes in genes associated with DNA damage repair, G2/M checkpoint regulation, and angiogenesis in human TNBC cells (Figures 2D-2F). Collectively, these findings indicate that ERK5 plays a multifaceted role in regulating immune-associated signaling and key cellular processes involved in cell cycle control and tumor progression.

### Loss of ERK5 impairs tumor growth and metastatic potential

**Figure 3:**
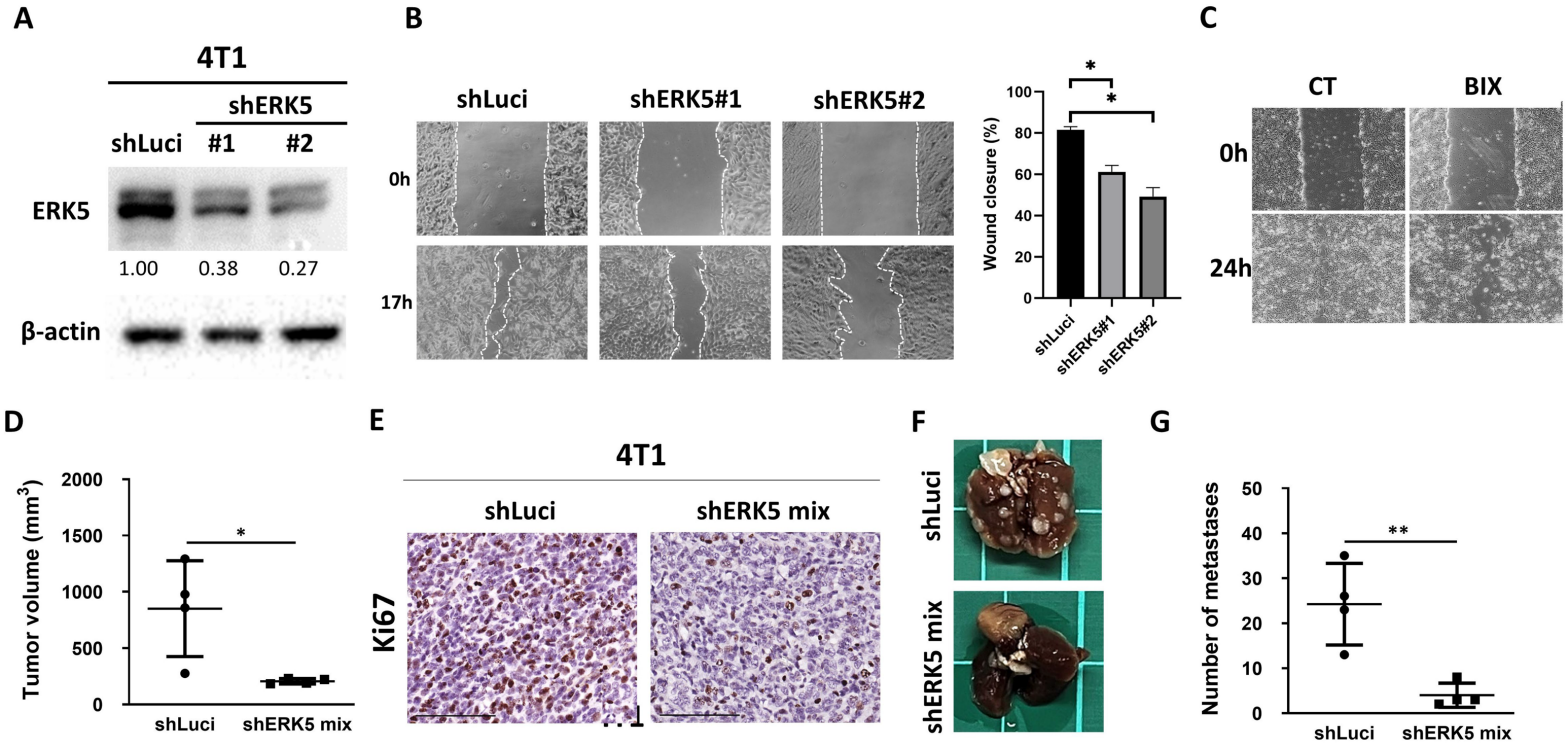
** Silencing ERK5 in 4T1 cells suppressed tumor growth, reduced lung metastases, and impaired cell migration.** (A) Western blot analysis confirming the successful establishment of stable ERK5-knockdown murine TNBC cell lines (4T1). β-actin was used as a loading control. (B-C) Wound healing assay for assessing the migratory capacity of 4T1 tumor cells. The experiments were repeated three times (*p < 0.05). (D) Tumor volume (mm^3^) at the orthotopic site at the endpoint of the experiment. (E) The tumors were collected and analyzed for Ki-67 using IHC. (F) Knockdown of ERK5 impaired the ability to induce distant lung metastasis. (G) Number of metastatic tumors in lungs. Data were presented as mean ± SD from four mice in each group. *, *p* < 0.05; **, *p* < 0.01. The scale bars in all images represent **100 μm**.

Given that these processes are closely linked to tumor cell plasticity and metastatic potential, we next sought to determine whether ERK5 also influences EMT, a critical program underlying tumor cell migration and invasion. Moreover, because immune- and angiogenesis-related pathways are strongly modulated by the tumor microenvironment, we extended our investigation to an immunocompetent murine breast cancer model to further elucidate the functional role of ERK5 in tumor progression. Western blot analysis confirmed successful, stable knockdown of ERK5 expression in 4T1 cells via RNAi (Figure 3A). After 17 hours of culture, ERK5-knockdown cells exhibited a significantly wider wound gap compared to control cells, indicating impaired migratory capacity (Figure 3B). Additionally, pharmacological inhibition of ERK5 in TNBC cells resulted in a comparable attenuation of cell migration (Figure 3C). Next, a syngeneic murine model was conducted using the 4T1 cell line and immunocompetent BALB/c mice to explore the functional role of ERK5 in breast cancer metastasis. Over the 27-day observation period, mice bearing ERK5-knockdown tumors exhibited significantly reduced tumor growth compared to the control group (p < 0.05) (Figure 3D). Immunohistochemical analysis further revealed that tumors from the ERK5-knockdown group exhibited a significantly lower Ki-67 positivity index, indicating decreased tumor cell proliferation (Figure 3E). Importantly, both macroscopic and histological evaluations revealed a significant reduction in the number of pulmonary metastatic nodules in the ERK5-knockdown group compared to the control group (Figures 3F and 3G). These findings collectively indicate that ERK5 contributes to both primary tumor growth and distal metastatic dissemination in this murine TNBC model.

### Suppression of ERK5 expression in murine TNBC cells leads to altered expression of proteins associated with EMT

**Figure 4:**
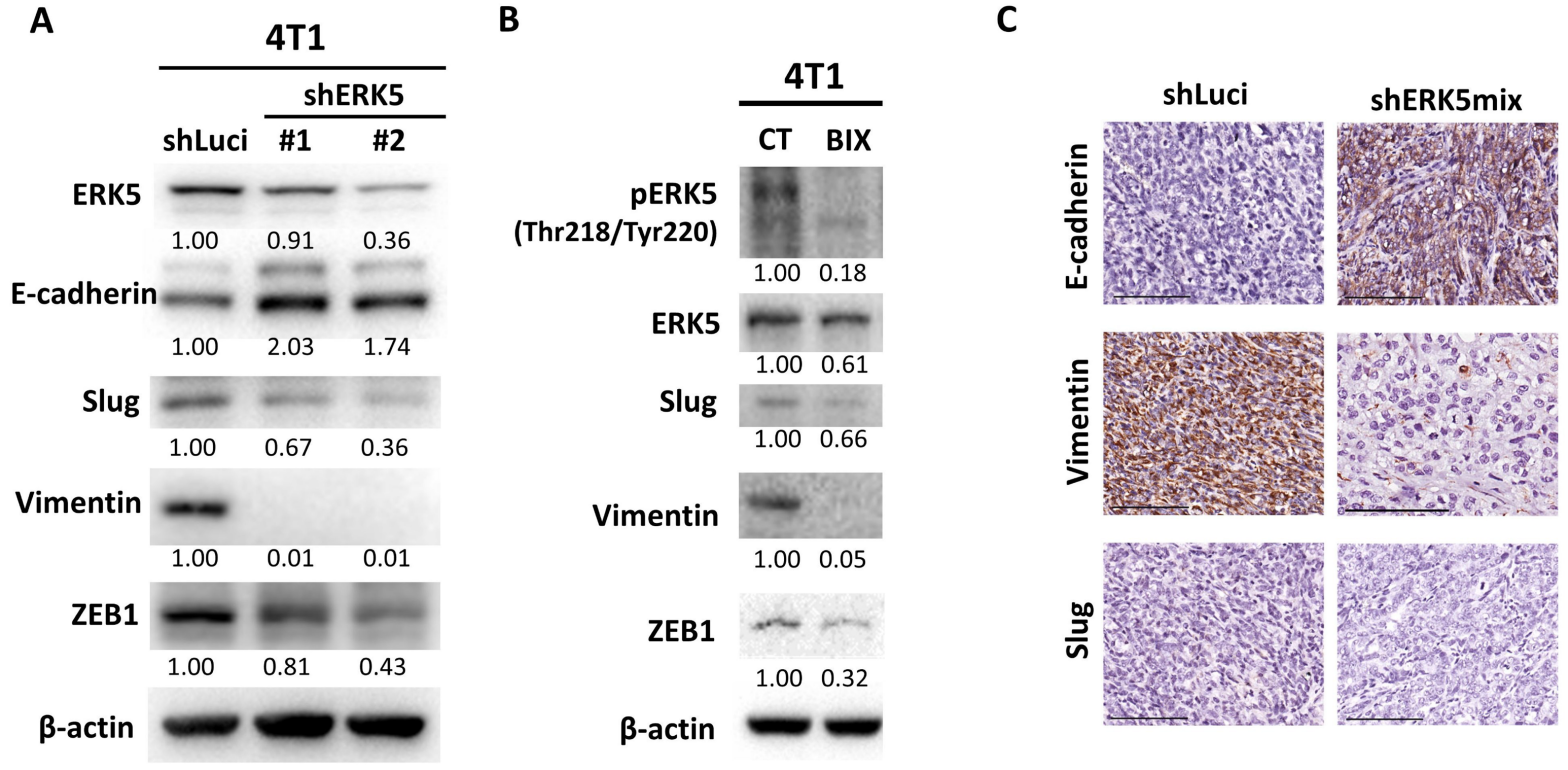
** ERK5 suppression affects proteins involved in epithelial-mesenchymal transition.** (A) Western blot analysis of the murine TNBC cell line (4T1-shERK5) compared with the control group (shLuci). β-actin was used as a loading control. (B) Western blot analysis comparing the ERK5 inhibitor-treated TNBC cell line (BIX) with the control group. β-actin was used as a loading control. (C) Expression levels of E-cadherin, Slug, vimentin, and ZEB1 in tumor tissue. The scale bars in all images represent **100 μm**.

Given that ERK5 expression influences the migratory capacity and the ability to form distal lung metastases in 4T1 murine TNBC cells (Figure 3), we next examined the molecular mechanisms underlying these phenotypes, with a particular focus on the potential role of ERK5 in EMT. Western blot analyses were conducted to compare the expression levels of EMT-related proteins between control 4T1 cells (shLuci) and two independent ERK5-knockdown cell lines (shERK5 #1 and #2). ERK5 suppression was associated with an increase in E-cadherin expression, along with a concomitant decrease in the expression of mesenchymal markers, including Slug, vimentin, and ZEB1 (Figure 4A). These results suggest that ERK5 may positively regulate EMT marker expression in TNBC cells. To evaluate whether pharmacological inhibition of ERK5 yields comparable effects, we treated 4T1 cells with the selective ERK5 inhibitor BIX02189. Inhibition of ERK5 catalytic activity reduced the levels of Slug, vimentin, and ZEB1 proteins, consistent with the molecular changes observed in ERK5-knockdown cells (Figure 4B). Immunohistochemical analysis of tumor tissues revealed a significant increase in E-cadherin IHC scores in tumors derived from ERK5-knockdown cells, coupled with a notable reduction in the proportion of vimentin-positive tumor cells (Figures 4C and Table 4). These observations were in close agreement with the *in vitro* findings. However, in contrast to the cell culture results, no significant difference in Slug expression was detected between control and ERK5-knockdown tumors *in vivo*, suggesting that ERK5-mediated regulation of Slug might be context-dependent or influenced by tumor microenvironmental factors.

### ERK5 regulates vimentin expression by modulating c-JUN binding to its promoter

**Figure 5:**
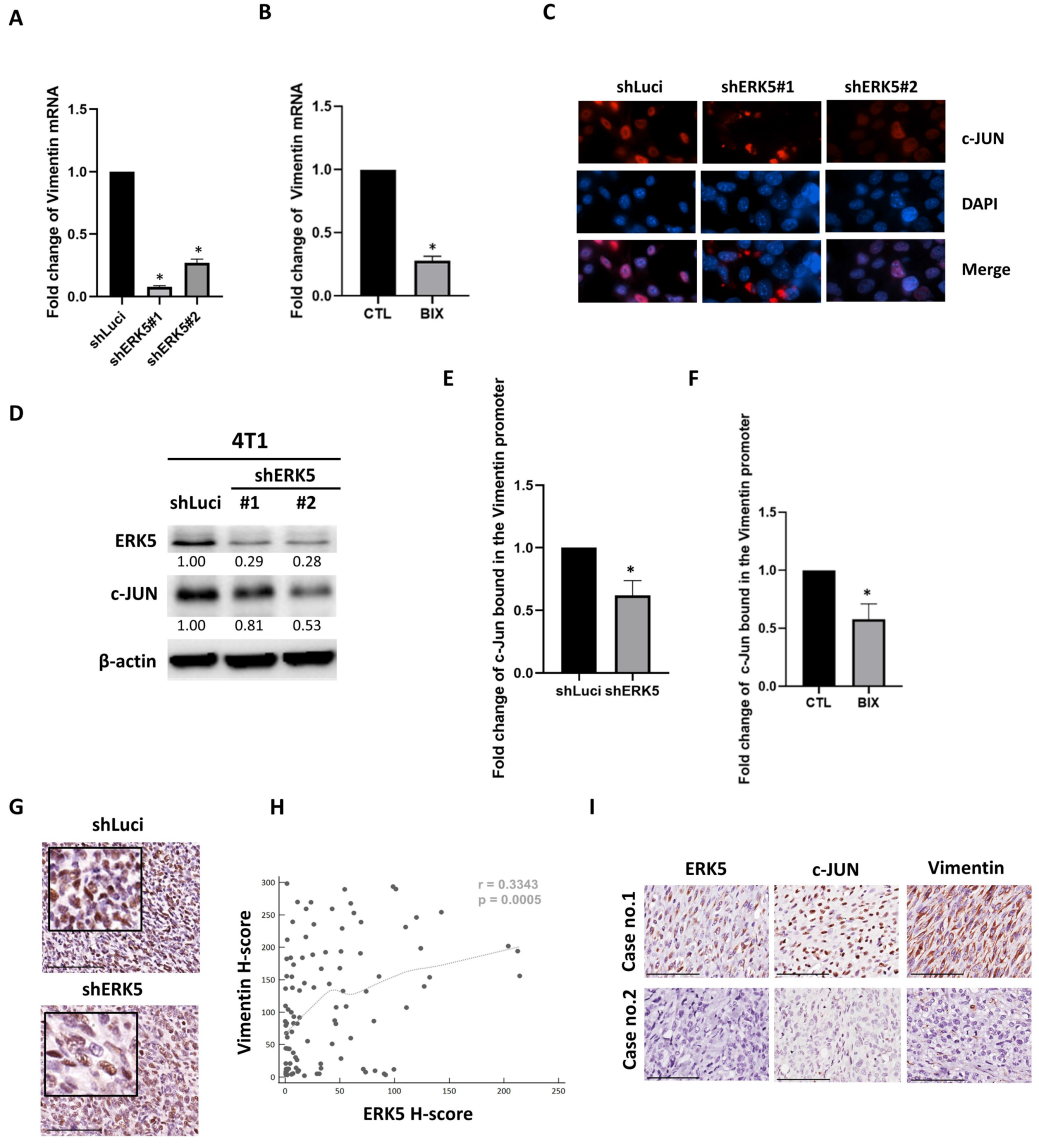
** RK5 promotes vimentin expression in TNBC by binding to the c-JUN promoter.** (A-B) Real-time PCR analysis of vimentin mRNA expression levels. (C) Representative immunofluorescence images showing c-JUN localization in control and ERK5-depleted cells. c-JUN is shown in red; nuclei are stained with DAPI (blue). Merged images demonstrate a clear reduction of nuclear c-JUN signal upon ERK5 knockdown compared with control cells. (D) Western blot showed a decrease in c-JUN expression in the ERK5 knockdown group. β-actin was used as a loading control. (E-F) Effect of ERK5 silencing on c-JUN (FOS::JUN) binding to the vimentin gene promoter, demonstrated by Chromatin Immunoprecipitation (ChIP) followed by qPCR. (G) Immunohistochemical staining for c-JUN in tumor tissue. (H) Pearson's correlation analysis was used to assess the correlation between ERK5 and vimentin expression. (I) ERK5 and vimentin protein expression levels in TNBC patient samples (representative images from cases 1 and 2 are shown). Data were presented as mean ± SD from four mice in each group. *, *p* < 0.05. The scale bars in all images represent **100 μm**.

Our *in vitro* and *in vivo* experiments consistently demonstrated that suppression of ERK5 expression in 4T1 murine TNBC cells led to a notable downregulation of vimentin protein levels (Figure 4). To investigate whether ERK5 modulates vimentin expression at the transcriptional level, we first performed quantitative real-time PCR to assess vimentin mRNA levels in ERK5-knockdown 4T1 cell lines (shERK5 #1 and #2) compared to control cells (shLuci). As shown in Figure 5A, ERK5 silencing significantly reduced vimentin mRNA expression. Consistently, pharmacological inhibition of ERK5 with BIX02189 also decreased vimentin transcript levels (Figure 5B).

Promoter analysis using the Eukaryotic Promoter Database identified five putative c-JUN (FOS::JUN) binding sites within the -1000 to +100 bp upstream region of the murine vimentin promoter, specifically at positions -188, -685, -687, -699, and -701. To investigate whether ERK5 regulates the nuclear localization of c-JUN, we examined c-JUN distribution following ERK5 depletion. Immunofluorescence analysis revealed that cells with reduced ERK5 expression displayed a marked decrease in nuclear c-JUN signal compared with control cells (Figure 5C). In addition, western blot data showed that ERK5 knockdown resulted in a decrease in c-JUN expression (Figure 5D). Given that vimentin expression was markedly reduced upon ERK5 knockdown and that AP-1 transcription factors are well-established regulators of EMT-related genes, we hypothesized that ERK5 may regulate vimentin expression through modulation of c-JUN-dependent transcriptional activity. To test this possibility, we next examined whether ERK5 influences c-JUN expression and its binding to the vimentin promoter. ChIP analysis revealed that ERK5 knockdown, either via RNA interference (4T1-shERK5) or pharmacological inhibition (BIX02189), significantly reduced c-JUN binding to the vimentin promoter compared with controls (Figures 5E-5F). Notably, ERK5 silencing decreased c-JUN protein expression in tumor tissues from murine TNBC models (Figure 5G).

Finally, to assess the clinical relevance of our findings, we examined ERK5 and vimentin protein expression in a cohort of 104 human TNBC tumor samples from E-Da Healthcare Group Hospitals and Kaohsiung Medical University Chung-Ho Memorial Hospital using immunohistochemistry and H-score quantification. Chi-square analysis revealed a statistically significant association between ERK5 and vimentin expression levels (p < 0.05), while Pearson's correlation analysis demonstrated a moderate positive correlation between the two proteins (r = 0.3343, p = 0.0005) (Figure 5H). Additionally, ERK5 expression showed a significant positive correlation with c-JUN expression. A similar trend was observed between c-JUN and vimentin expression, though it did not reach statistical significance. The correlations among the expression levels of ERK5, c-JUN, and vimentin are shown in Figure 5I and Table 5.

## Discussion

In this study, we established a clear association between ERK5 expression in tumor cells and patient prognosis in TNBC. Silencing ERK5 in the highly metastatic human TNBC cell line IV2-1 (MDA-MB-231 subline) led to significant alterations in the expression of genes involved in immune response, DNA damage repair, the G2/M checkpoint, and angiogenesis. *In vivo*, ERK5 inhibition in the murine TNBC cell line, 4T1, resulted in a marked decrease in the size and proliferative activity of primary subcutaneous tumors. This suppression notably diminished the cell's migratory ability, reduced EMT-related proteins such as vimentin, and reduced the incidence of distant lung metastases. Furthermore, we found that ERK5 in TNBC cells regulates vimentin expression by modulating c-Jun binding to the vimentin promoter, thereby affecting subsequent changes in cell properties. Using clinical samples from patients with TNBC, we confirmed a significant correlation between ERK5 expression and vimentin levels in breast cancer cells. Based on these findings, we demonstrated for the first time that the ERK5-c-JUN-vimentin signaling axis in TNBC cells promotes EMT and distant tumor metastasis, influencing disease progression and prognosis.

Our findings are supported by previous studies indicating that ERK5 plays a role in EMT regulation. For example, Hoang *et al*. 28 reported that utilizing CRISPR/Cas9 technology to knock out ERK5 expression in the TNBC cell lines, Hs-578T and MDA-MB-231, caused the tumor cells to transition from a mesenchymal spindle-like morphology to a more rounded epithelial-like phenotype, and increased the expression of the epithelial marker, E-cadherin. Concurrently, it suppressed the expression of mesenchymal-associated genes, including FRA-1, ZEB1, JAG1, SNAI1, VIM, and MMP2. Through *in vitro* and *in vivo* experiments, the authors further demonstrated that suppression of ERK5 impaired tumor cell migration, reduced the number of lung metastases, and altered the composition and structure of the extracellular matrix. However, the detailed molecular mechanisms underlying these changes were not explored or elucidated in this study. In contrast, Xu *et al*. found that inhibiting ERK5 expression in MDA-MB-231 promoted the expression of the epithelial marker E-cadherin and decreased the expression of the EMT-related protein Slug. However, this inhibition did not alter the expression of N-cadherin, MMP2, or vimentin. The underlying molecular mechanism was linked to phosphorylation and subsequent activation of focal adhesion kinase at Tyrosine 397 29.

In most cases, ERK5 is phosphorylated and activated at its TEY motif by its specific upstream activator MEK5. ERK5 may also be phosphorylated at serine and threonine residues on its C-terminal tail by CDK1 and/or ERK1/2. Once activated, ERK5 translocates to the nucleus, where it exerts dual functions in gene regulation by directly phosphorylating and activating transcription factors and by serving as a transcriptional cofactor that influences gene expression 14, 30-32. Therefore, it is generally believed that drugs that affect ERK5 catalytic activity (e.g., BIX02189) will block ERK5's regulation of downstream molecules and signaling pathways 33. Previous studies have shown that several ERK5-transcription factors signaling pathways, including the ERK5-AP-1 axis and the MEK5/ERK5/NF-κB signaling pathway, are closely linked to tumor invasion and metastasis 12, 19, 34-37. Liu *et al*. 38 studied curcumin and urothelial cancer and observed that benzidine stimulation increased ERK5 phosphorylation in urothelial cells. Phosphorylated ERK5, in turn, activates AP-1 (comprising c-JUN and c-FOS), leading to morphological changes in urothelial cells, enhanced migratory and invasive capacities, and altered expression of EMT-related proteins. Similarly, Zhang *et al*. 39, reported comparable findings in their study on renal cell carcinoma and curcumin. Moreover, Sun *et al*. 40 revealed that inhibiting the ERK5-AP-1 pathway could counteract the benzidine-induced EMT in urothelial carcinoma cells. However, the molecular mechanisms by which ERK5 promotes EMT and distant metastasis in TNBC cells remain unclear, particularly whether they involve the AP-1 family, including c-JUN or c-FOS, as observed in urothelial and renal cancers. To date, no studies have explored this relationship. To our knowledge, this is the first study to demonstrate a regulatory relationship between ERK5, c-JUN, and vimentin in TNBC cells. We also found that this regulatory pathway influences EMT and metastatic processes, suggesting that our findings may identify novel targets for developing future therapies against TNBC.

This study has certain limitations that warrant consideration. While our data support the involvement of the ERK5/c-JUN/vimentin axis in regulating EMT and metastasis in the 4T1-BALB/c murine TNBC model, and we observed a significant correlation between ERK5 and vimentin expression in clinical TNBC specimens, the functional relevance of this pathway in human TNBC cells remains to be further explored. Given the biological differences between murine and human systems, additional validation using human TNBC cell lines and models will be essential to confirm the broader applicability of our findings. Moreover, ERK5 was found to regulate additional EMT-related markers, including E-cadherin and Slug, in both *in vitro* and *in vivo* murine models, further supporting a broader role for ERK5 in EMT regulation. Nevertheless, whether these regulatory effects are also present in human TNBC remains to be determined, and further mechanistic studies are needed to clarify the underlying molecular basis. In the present study, although ERK5 expression was significantly associated with PFS, it was not significantly associated with OS or DMFS. Several explanations may account for this discrepancy. First, during distant colonization and disease progression, compensatory activation of alternative signaling pathways may attenuate ERK5's contribution to later-stage clinical outcomes. Second, OS is influenced not only by the biological behavior of the primary tumor but also by subsequent systemic therapies administered after initial progression, potentially obscuring the direct prognostic impact of ERK5 expression. In this context, Broglio and Berry 41 demonstrated that the discordance between PFS and OS in metastatic cancer trials is largely determined by survival post-progression (SPP). Because OS can be considered the sum of PFS and SPP, it is substantially affected by treatments received after progression. In contrast, PFS more directly reflects early disease control and the effects of first-line therapy. In addition, intratumoral heterogeneity and branched tumor evolution may further contribute to variable clinical outcomes after progression 42. Therefore, the absence of a statistically significant association between ERK5 expression and OS or DMFS does not necessarily preclude its clinical relevance, particularly in settings with a prolonged median SPP.

Our results showed that ERK5 knockdown reduced c-JUN expression, nuclear localization, and binding to the vimentin promoter, suggesting that ERK5 participates in regulating the c-JUN/vimentin axis. Nevertheless, the present study did not determine whether ERK5 regulates c-JUN directly, such as through phosphorylation, or indirectly via upstream signaling pathways. Notably, previous studies have demonstrated that ERK5 signaling can regulate downstream transcription factors, including members of the AP-1 family and c-FOS 12, 14, supporting the biological plausibility of ERK5-mediated transcriptional regulation. Accordingly, our data support a functional link between ERK5 and c-JUN-dependent vimentin expression, while the detailed molecular basis of this regulation remains to be clarified.

Taken together, these findings suggest that ERK5 contributes to tumor progression and EMT-related phenotypes in TNBC. PFS may more readily reflect its prognostic significance than by OS or DMFS. Further studies are warranted better to define the context-dependent role of ERK5 in TNBC progression and to determine whether ERK5 may serve as a meaningful therapeutic target in specific clinical settings.

## Conclusion

In summary, IHC analysis of TNBC clinical specimens revealed a strong association between ERK5 expression, poor prognosis, and vimentin levels. In human TNBC cells, ERK5 knockdown altered the expression of genes related to immune response, DNA repair, G2/M checkpoint control, and angiogenesis. *In vitro* and *in vivo* experiments further showed that ERK5 regulates vimentin expression, tumor cell migration, EMT-related proteins, and lung metastasis, partly by modulating c-JUN binding to the vimentin promoter. While these findings suggest an ERK5-c-JUN-vimentin regulatory axis, further studies are needed to confirm its role in TNBC metastasis and EMT. Identifying additional molecules involved in this pathway may help uncover new therapeutic targets to improve treatment outcomes in TNBC.

## Supplementary Material

Supplementary figure.

## Figures and Tables

**Table 1:** Baseline characteristics of study cohort (n=117).

Parameters	ERK5, n (%)			*p* value
	Low	Medium	High	
Age (year)	54 (19-93)	55 (23-90)	53(26-88)	0.2427
**TIL**				0.0517
1	14 (27.45)	19 (43.18)	11 (50.00)	
2	7 (13.73)	12 (27.27)	3 (13.64)	
3	15 (29.41)	5 (11.36)	6 (27.27)	
4	15 (29.41)	8 (18.18)	2 (9.09)	
**Tumor size**				0.5970
< 2cm	15 (29.41)	9 (20.45)	6 (27.27)	
≥ 2cm	36 (70.59)	35 (79.55)	16 (72.73)	
**Tumor type**				0.7259
Non-IDC	11 (21.57)	7 (15.91)	5 (22.73)	
IDC	40 (78.43)	37 (84.09)	17 (77.27)	
**Histologic grade**				0.3878
I	1 (1.96)	1 (2.27)	2 (9.09)	
II	20 (39.22)	13 (29.55)	9 (40.91)	
III	30 (58.82)	30 (68.18)	11 (50.00)	
**T status**				0.5131
T1/2	43 (84.31)	33 (75.00)	18 (81.82)	
T3/4	8 (15.69)	11 (25.00)	4 (18.18)	
**N status**				0.6723
N0	31 (60.78)	25 (56.82)	15 (68.18)	
N1/2/3	20 (39.22)	19 (43.18)	7 (31.82)	
**M status**				0.3801
M0	48 (94.12)	38 (86.36)	21 (95.45)	
M1	3 (5.88)	6 (13.64)	1 (4.55)	
**LVI**				0.9849
Negative	24 (47.06)	21 (47.73)	10 (45.45)	
Positive	27 (52.94)	23 (52.27)	12 (54.55)	
**Dermal invasion**				0.8672
Negative	42 (87.50)	38 (86.36)	20 (90.91)	
Positive	6 (12.50)	6 (13.64)	2 (9.09)	
**Perineural invasion**				0.3516
Negative	40 (78.43)	39 (88.64)	17 (77.27)	
Positive	11 (21.57)	5 (11.36)	5 (22.73)	
**Recurrence**				**0.0150**
Absent	48 (94.12)	42 (95.45)	16 (72.73)	
Present	3 (5.88)	2 (4.55)	6 (27.27)	
**Survival status**				0.1353
Survived	42 (82.35)	29 (65.91)	18 (81.82)	
Died	9 (17.65)	15 (34.09)	4 (18.18)	

Abbreviations: TIL, tumor-infiltrating lymphocytes; IDC, invasive ductal carcinoma (IDC); LVI, lymphovascular invasion. p<0.05 was considered statistically significant.

**Table 2:** Pearson analysis of the relation between patients' prognosis and ERK5 (n=117).

Parameters	Recurrence (Y/N)	M status (Y/N)	Patientsurvival (Y/N)	Tumorprogression (Y/N)
ERK5	0.22360	0.01951	0.05182	0.21591
*p value*	**0.0154**	0.8346	0.5790	**0.0194**

**Table 3:** Univariate and multivariable Cox regression analyses of progression-free survival according to ERK5 expression and clinicopathological variables in patients with TNBC (n = 117).

Parameters	Comparison	Univariate Analysis			Multivariable Analysis	
		HR (95%CI)	p-value	HR (95%CI)		p-value
ERK5	Medium	1.708 (0.766-3.807)	0.1904	2.008 (0.856-4.711)		0.1089
	High	2.701 (1.124-6.495)	**0.0264**	2.875 (1.152-7.174)		**0.0236**
Size	≥ 2cm	3.902 (1.194-12.751)	**0.0242**	3.182 (0.946-10.700)		0.0614
Tumor type	IDC	2.920 (0.891-9.568)	0.0768	3.123 (0.918-10.627)		0.0683
Grade	II	0.820 (0.188-3.582)	0.7916	1.008 (0.208-4.894)		0.9920
	III	0.431 (0.099-1.874)	0.2616	0.609 (0.121-3.050)		0.5461
T	T3/4	4.338 (2.124-8.860)	**< 0.0001**			
N	N1/2/3	1.695 (0.869-3.309)	0.1217			
M	M1	5.044 (2.240-11.360)	**< 0.0001**			
LVI	Positive	1.594 (0.808-3.142)	0.1784			
Dermal invasion	Positive	3.948 (1.829-8.522)	**0.0005**			
Perineural invasion	Positive	1.516 (0.708-3.246)	0.2836	1.507(0.639-3.553)		0.3488
						

**Table 4:** The correlation among ERK5, c-JUN, and vimentin expression levels.

Parameter	ERK5, n (%)		*p* value
	High	Low	
**E-cadherin**			**< 0.05**
High	0 (0)	3 (75)	
Low	4 (100)	1 (25)	
**Vimentin**			**< 0.05**
High	4 (100)	1 (25)	
Low	0 (0)	3 (75)	
**Parameter**	**c-JUN, n (%)**		***p* value**
	**High**	**Low**	
**Slug**			0.1266
High	2 (50)	0 (0)	
Low	2 (50)	4 (100)	

**Table 5:** The correlation among ERK5, c-JUN, and vimentin expression levels.

	ERK5, n (%)				
Vimentin	Low	Medium	High	Total	*p* value
Low	34	15	3	52 (50.0)	**0.0007**
High	16	23	13	52 (50.0)	
	50 (48.1)	38 (36.5)	16 (15.4)		
	**ERK5, n (%)**				
**c-JUN**	**Low**	**Medium**	**High**	**Total**	***p* value**
Low	26	23	3	52 (50.0)	**0.0182**
High	24	15	13	52 (50.0)	
	50 (48.1)	38 (36.5)	16 (15.4)		
	**c-JUN, n (%)**				
**Vimentin**	**Low**		**High**	**Total**	***p* value**
Low	31		21	52 (50.0)	0.0510
High	21		31	52 (50.0)	
	52 (50.0)		52 (50.0)		

## Data Availability

The datasets used and/or analyzed during the current study are available from the corresponding author upon reasonable request.

## Abbreviations

AP-1: Activator protein 1
BMK1: Big mitogen-activated protein kinase 1
cDNA: Complementary DNA
ChIP: Chromatin immunoprecipitation
c-FOS: Cellular FBJ murine osteosarcoma viral oncogene homolog
c-MYC: Cellular myelocytomatosis viral oncogene homolog
DMFS: Distant metastasis-free survival
EMT: Epithelial-to-mesenchymal transition
ER: Estrogen receptor
ERK5: Extracellular signal-regulated kinase 5
JNK1/2/3: c-Jun N-terminal Kinase (JNK)1/2/3
GSEA: Gene set enrichment analysis
GSK-3β: Glycogen synthase kinase 3 beta
HER2: Human epidermal growth factor receptor 2
H&E: Hematoxylin and eosin
IHC: Immunohistochemical
MAPK7: Mitogen-activated protein kinase 7 (MAPK7)
MEF2: Myocyte enhancer factor 2
NF-κB: Nuclear factor kappa beta
PR: Progesterone receptor
qPCR: Quantitative real-time polymerase chain reaction
TGF-β: Transcription growth factor beta
RNAi: RNA interference
RFS: Relapse-free survival
TNBC: Triple-negative breast cancer
TWIST: Twist family BHLH transcription factor
ZEB1: Zinc finger E-box binding homeobox
