# Supplementary material for: Targeting the Extracellular Signal-Regulated Kinase 5-Cellular Jun-Vimentin Axis to Inhibit Epithelial-Mesenchymal Transition and Metastasis in Patients with Triple-Negative Breast Cancer
Source: Int J Med Sci. 2026 Jun 17;23(8):2524–39. doi: 10.7150/ijms.131682 (PMC13411615; doi:10.7150/ijms.131682)
Supplement: Supplementary file 1 — Supplementary figure. [file ijmsv23p2524s1.pdf]

Figure S1.

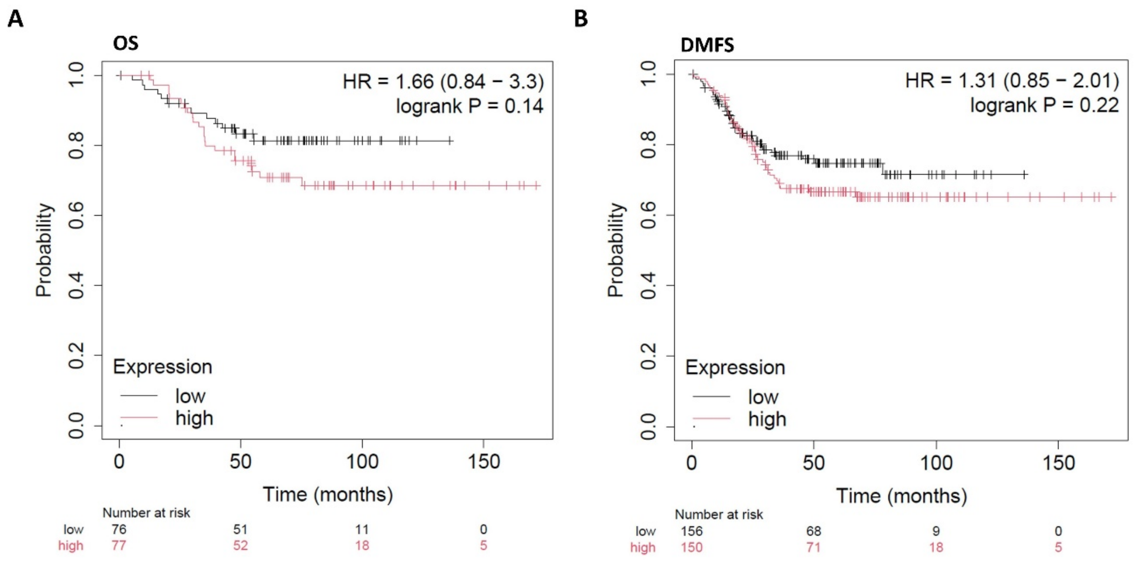

Figure S1. Kaplan-Meier survival analysis based on messenger RNA (mRNA) expression data (probe ID: 207292_s_at) obtained from the Kaplan-Meier Plotter database showing (A) overall survival (OS) and (B) distant metastasis-free survival (DMFS).
